# Cdk5 modulates orofacial pain through P2X2/3 purinergic receptor-mediated signaling in trigeminal neurons

**DOI:** 10.3389/fphar.2026.1767160

**Published:** 2026-05-04

**Authors:** Rodrigo Sandoval, Nicolás Pinto-Pardo, Camila Duran, Patricio Castro, Gabriela Rossi, Christian González-Billault, María Pertusa, Rodolfo Madrid, Claudio Coddou, Elías Utreras

**Affiliations:** 1 Department of Biology, Faculty of Science, Universidad de Chile, Santiago, Chile; 2 Dentistry, Department of Dental Sciences, Faculty of Life Sciences, Universidad Viña del Mar, Viña del Mar, Chile; 3 Department of Biomedical Sciences, Faculty of Medicine, Universidad Católica del Norte, Coquimbo, Chile; 4 Department of Physiology, Faculty of Biological Sciences, Universidad de Concepción, Concepción, Chile; 5 Department of Neurosciences, Faculty of Medicine, Universidad de Chile, Santiago, Chile; 6 Geroscience Center for Brain Health and Metabolism, Santiago, Chile; 7 The Buck Institute for Research on Aging, Novato, CA, United States; 8 Department of Biology, Faculty of Chemistry and Biology, Universidad de Santiago de Chile, Santiago, Chile

**Keywords:** ATP-gated channels, Cdk5, p35, phosphorylation, purinergic signaling

## Abstract

**Introduction:**

Cyclin-dependent kinase 5 (Cdk5) plays a critical role in pain transmission by phosphorylating key nociceptive proteins. One of its recently identified substrates, the P2X2 subunit, is part of the P2X2/3 purinergic receptor (P2X2/3R), which is implicated in nociceptive signaling. However, the specific contribution of Cdk5 to purinergic receptor-mediated pain responses remains unclear. This study aimed to investigate the role of Cdk5 in pain responses elicited by α,β-methylene ATP (α,β-meATP), an agonist of P2X2/3R and P2X3R.

**Methods:**

Both pharmacological inhibition and genetic loss-of-function approaches were used to assess the role of Cdk5. Calcium (Ca^2+^) imaging was performed in cultured murine trigeminal ganglion (TG) neurons to characterize α,β-meATP-evoked responses. Surface biotinylation assays in HEK293 cells were used to assess P2X2 receptor membrane expression. *In vivo*, facial pain was induced by injecting α,β-meATP into the whisker pad of mice. Behavioral responses were evaluated in conditional Cdk5 knockout mice targeting sensory neurons and in control littermates.

**Results:**

In TG neuron cultures, α,β-meATP evoked both fast and slow Ca^2+^ transients, which were sensitive to P2X3 or P2X2/3 channel blockade. Inhibition or genetic loss-of-function of Cdk5 predominantly accelerated the decay of slow responses, with minimal effects on fast responses. In HEK293 cells, Cdk5 activation did not alter the surface expression of P2X2 receptors. *In vivo*, α,β-meATP induced facial pain-like behaviors, including grooming and head flinching, which were significantly reduced in conditional Cdk5 knockout mice compared to controls.

**Discussion:**

These findings indicate that Cdk5 modulates orofacial pain by regulating purinergic receptor-mediated signaling in trigeminal neurons. Specifically, Cdk5 appears to influence the functional properties of P2X2-containing channels, most likely P2X2/3 heteromeric receptors, without affecting their membrane expression. This modulation of receptor kinetics may underlie the observed changes in nociceptive behavior, highlighting Cdk5 and P2X receptors as a potential target for pain modulation.

## 1 Introduction

Pain is mediated by a complex neural network that encodes various noxious stimuli in the periphery and conveys this information to the central nervous system (Basbaum et al., 2009). The trigeminal ganglion (TG) is a unique structure where afferent nerve fibers converge to relay nociceptive signals originating from various orofacial tissues including the meninges, cornea, dental pulp, and temporomandibular joint, among others. Importantly, the physiology of orofacial pain exhibit unique characteristics that distinguish it from somatic pain experienced in other parts of the body (Bereiter et al., 2008; Hargreaves, 2011; Megat et al., 2019). Multiple receptors and ion channels expressed on the peripheral terminals of nociceptors are responsible for transducing physical and chemical stimuli into electrical signals that are relayed to the trigeminal spinal nucleus (Van der Cruyssen and Politis, 2018; Bista and Imlach, 2019). Inflammation is a hallmark of many painful conditions and promotes the development of pain hypersensitivity (Basbaum et al., 2009; Bista and Imlach, 2019). This process often involves the activation of kinases that phosphorylate ion channels and other proteins, thereby increasing the excitability of nociceptors (Basbaum et al., 2009; Ji et al., 2014; Kandasamy and Price, 2015). Cyclin-dependent kinase 5 (Cdk5) is a key kinase implicated in pain signaling (Utreras et al., 2009b). It becomes active upon binding to its activators, p35 or p39, which are predominantly expressed in postmitotic cells (Hellmich et al., 1992; Tsai et al., 1994; Dhavan and Tsai, 2001). Once activated, Cdk5 phosphorylates proline-directed serine/threonine residues in a wide range of substrates (Su and Tsai, 2011). It was demonstrated that Cdk5 phosphorylates the transient receptor potential vanilloid 1 (TRPV1) and ankyrin (TRPA1) channels, enhancing Ca^2+^ influx in sensory neurons (Pareek et al., 2007; Utreras et al., 2009a; Utreras et al., 2012; Jendryke et al., 2016; Rozas et al., 2016; Hall et al., 2018; Sulak et al., 2018). In addition, Cdk5 regulates TRPV1 trafficking to the plasma membrane through multiple mechanisms (Xing et al., 2012; Liu et al., 2015; Liu et al., 2019). These molecular mechanisms align with behavioral data from murine models with altered Cdk5 function, which show consistent pain phenotypes (Wang et al., 2005; Pareek et al., 2006; Pareek et al., 2007; Utreras et al., 2009a; Prochazkova et al., 2013; Jendryke et al., 2016; Rozas et al., 2016; Hall et al., 2018). More recently, we reported that Cdk5 phosphorylates purinergic P2X2 subunit (Coddou et al., 2017). Purinergic P2X receptors (P2XR) are ATP-gated ion channels that allow the influx of Na^+^, and Ca^2+^ (Coddou et al., 2011; Puchalowicz et al., 2014). In mammals, seven P2X receptor subunits (P2X1–7) can assemble into functional homo- and heterotrimeric channels, with their distribution influencing both pharmacological and electrophysiological properties (North, 2002; Coddou et al., 2011). There are different splice variants of P2X2, with P2X2a being the full-length receptor and P2X2b and P2X2e being shorter variants in mice (Coddou et al., 2011). Interestingly, P2X2 and P2X3 are the predominant subunits expressed in trigeminal sensory neurons, and they form either fast-desensitizing P2X3R homotrimers or slow-desensitizing P2X2/3R heterotrimers, both of which are involved in pain signaling (Cockayne et al., 2005; Staikopoulos et al., 2007). We previously found that Cdk5 phosphorylates P2X2aR at threonine 372, resulting in reduced desensitization of receptors containing this subunit (Coddou et al., 2017). In contrast to the extensively studied P2X3R, the specific contribution of P2X2-containing receptors, particularly in heteromeric P2X2/3 configurations, to nociceptive signaling remains poorly defined, especially at the level of intracellular regulatory mechanisms such as kinase-dependent modulation.

This study examines the regulatory role of Cdk5 in purinergic signaling and investigates its potential implications for modulating orofacial pain. Using Ca^2+^ imaging, we demonstrate that Cdk5 modulates the kinetics of both P2X3 and P2X2/3 receptors. Furthermore, intradermal injection into the whisker pad of α,β-meATP, a non-selective agonist of P2X3R and P2X2/3R (Burnstock and Kennedy, 1985), elicited facial pain-like behaviors, such as grooming and flinching in mice. Importantly, these responses were significantly reduced in conditional Cdk5 knockout (Cdk5 cKO) mice in sensory neurons compared to control littermates. Together, our results suggest that Cdk5 increases P2X2/3R-mediated purinergic signaling, contributing to orofacial pain. This adds a new hub to the molecular pathways modulated by Cdk5 in nociception, supporting further investigation into Cdk5 as a potential therapeutic target for pain management.

## 2 Methods

### 2.1 Culture and transfection of HEK293 cells

HEK293 cells (ATCC# CRL-1573) were maintained in Dulbecco’s Modified Eagle Medium (DMEM) supplemented with 10% fetal bovine serum (FBS) and penicillin/streptomycin (Invitrogen, Carlsbad, CA). For surface biotinylation assays, HEK293 cells were seeded in 60-mm culture plates and transiently co-transfected for 24 h with 1 µg of rat P2X2aR-pIRES-GFP and 2 µg of either mouse CMV-p35 or pcDNA 3.1 empty vector using Lipofectamine 2000 reagent (Invitrogen, Carlsbad, CA). For patch-clamp experiments, HEK293 cells were plated in 24-well plates at a density of 1 × 10^5^ cells per well and transfected with 0.3 µg of either rat P2X2aR-pIRES-GFP, P2X2aR^T372A^-pIRES-GFP, or P2X2aR^T372D^-pIRES-GFP, along with 0.7 µg of either CMV-p35 or pcDNA 3.1 empty vector. After 24 h, the transfected cells were trypsinized and replated onto 6-mm poly-L-lysine–coated glass coverslips, where they were cultured for additional 24 h before undergoing electrophysiological recording.

### 2.2 Primary culture of mouse or rat trigeminal neurons

Trigeminal primary sensory neurons were cultured as described previously (Rozas et al., 2016; Piña et al., 2024). Briefly, TG were dissected from adult (2–8 month old) male and female wild type and Cdk5 cKO mice (C57BL6) or adult female rats (Sprague-Dawley) and incubated with collagenase XI (0.66 mg/mL) and dispase II (3 mg/mL) (Sigma-Aldrich, St. Louis, MO) in INC-mix solution (155 mM NaCl, 1.5 mM K_2_HPO_2_, 10 mM HEPES, 5 mM glucose, pH 7.4). Enzymatic digestion was carried out for 45 min at 37 °C in a 5% CO_2_ atmosphere, followed by an additional 10 min of digestion with DNase I (0.1 mg/mL; Roche Diagnostics, Indianapolis, IN) under the same conditions. Cellular debris was removed in two steps using a 70 μm cell strainer (BD Biosciences, San Jose, CA) and a 28%/12.5% Percoll density gradient (GE Healthcare, Little Chalfont, United Kingdom). Cells were cultured in Minimum Essential Medium (MEM) supplemented with 10% fetal bovine serum (FBS), 100 μg/mL penicillin/streptomycin, and MEM-vitamins (Invitrogen, Carlsbad, CA). Cells were plated onto 12-mm poly-L-lysine–coated glass coverslips and cultured for 2 days.

For Cdk5 kinase activity inhibition experiments, cultured TG neurons were treated with roscovitine (20 μM; Sigma-Aldrich, St. Louis, MO) for 6 h prior to Ca^2+^ imaging. For inhibition of responses mediated by P2X3-containing channels we used two well-known antagonist: A-317491 (3 μM; Sigma-Aldrich, St. Louis, MO) added to the primary cultures 45 min prior to Ca^2+^ imaging and that was also present during α,β-meATP stimulation in mouse TG neurons, and Gefapixant (1 μM; Ann Arbor, MI) that was incubated for 10 min before α,β-meATP stimulation for Ca^2+^ imaging in rat TG neurons. All animal procedures were conducted in accordance with the National Institutes of Health Guidelines for the Care and Use of Laboratory Animals and approved by the Bioethical Committee of Universidad Católica del Norte (Coquimbo, Chile) and the Ethics Committee of the Biology Department, Faculty of Sciences, Universidad de Chile (Santiago, Chile).

### 2.3 Calcium imaging in cultured TG neurons

Cultured TG neurons were incubated with Fluo-4 AM (3 μM, Molecular Probes, Eugene, OR) for 30 min at 37 °C. After loading, cells were washed twice with phosphate-buffered saline (PBS) and incubated for an additional 30 min at 37 °C. Cells were then mounted in a perfusion chamber placed on the stage of a Zeiss LSM800 confocal microscope (Carl Zeiss Microscopy GmbH, Jena, Germany), maintained at 22 °C–24 °C. The purinergic agonist α,β-meATP (100 μM; Sigma-Aldrich, St. Louis, MO) was added to the bath solution. Cells were briefly illuminated using minimal laser power (≤0.5%) and scan speeds of 6–7, controlled by ZEN 2.1 software (Carl Zeiss Microscopy GmbH, Jena, Germany). Regions of interest (ROIs) were selected on neuronal somas exhibiting Fluo-4 fluorescence (excitation: 480 nm; emission: 510 nm), typically in fields containing more than 20 cells. Images were acquired every 2 s for a total duration of 12 min, using 16-bit imaging (0–65,000 fluorescence units) and a highly sensitive GaAsP detector (Carl Zeiss Microscopy GmbH). The same protocol was used for experiments using the P2X3 antagonist Gefapixant.

For the experiments using TG cultures derived from Cdk5 cKO and control littermate mice, neurons were incubated with 5 μM Fura-2AM (Invitrogen) in standard extracellular solution containing 140 mM NaCl, 3 mM KCl, 1.3 mM MgCl_2_, 2.4 mM CaCl_2_, 10 mM glucose, and 10 mM HEPES, pH 7.4 (adjusted with NaOH, 297 mOsm/kg), supplemented with 0.02% Pluronic acid (Thermo Fisher, Invitrogen, Waltham, MA, United States). Cultures were incubated for 45 min at 37 °C in the dark. Fluorescence was measured using an inverted Nikon Eclipse Ti-U microscope equipped with a 12-bit cooled ORCA C8484–03G02 CCD camera (Hamamatsu, Hamamatsu City, Japan). Fura-2 was excited at 340 nm and 380 nm using a Polychrome V monochromator (Till Photonics, Munich, Germany) with 50 ms exposure times. Emitted fluorescence was collected through a 510 nm long-pass filter. F340/380 ratios were sampled at 1 Hz and monitored in real time using HCImage v1.2 software (Hamamatsu, Hamamatsu City, Japan). Coverslips containing cultured trigeminal neurons were placed in a microchamber and continuously perfused with solutions maintained at approximately ∼33 °C. Bath temperature was monitored using an IT-18 Type T thermocouple connected to a Physitemp BAT-12 microprobe thermometer (Physitemp Instruments, Clifton, NJ, United States). Temperature data were using a Digidata 1440A AD converter with Clampex 10 software (Molecular Devices, San Jose, CA, United States). In these experiments, a 3-min baseline recording was followed by a 3-min application of 10 µM α,β-meATP. Finally, a 30 mM KCl depolarizing stimulus was applied at the end of the experiment. The same protocol was used for experiments using the P2X3-containing channels antagonist A-317491.

Ca^2+^ transients were defined as responses with amplitudes at least twice the baseline noise level. Data acquisition and analysis were performed offline using ImageJ 1.46 (NIH, Bethesda, MD) and GraphPad Prism version 7 (GraphPad Software, San Diego, CA). These Ca^2+^ transients were classified by decay kinetics, and the interval between the peak fluorescence (N_0_) and the plateau (P) was manually selected and fitted to either a one-phase or two-phase exponential decay function to obtain the time constant tau (τ):

One-phase decay equation:

$$Y=N_{0}\times e^{\left(-\frac{X}{\tau }\right)}+P$$

Two-phase decay equation:

$$Y=N_{0}1\times e^{\left(-\frac{X}{\tau \;fast}\right)}+N_{0}2\times e^{\left(-\frac{X}{\tau \;slow}\right)}+P$$

### 2.4 Current measurements in HEK293 cells

Patch-clamp experiments were performed at room temperature using the whole-cell configuration, with a holding potential (V_hold_) of −60 mV. Currents were recorded using an Axopatch 200B patch-clamp amplifier (Molecular Devices, Sunnyvale, CA) and filtered at 2 kHz using a low-pass Bessel filter. Data acquisition and storage were performed with pClamp 10 software via an Axon Digidata 1440A AD converter (Molecular Devices, Sa Jose, CA). Patch pipettes (4–6 MΩ) were pulled from GC150F-7.5 glass capillaries (Harvard Apparatus, Holliston, MA) and filled with intracellular solution containing: 130 mM NaCl, 10 mM HEPES, 10 mM EGTA (305 mOsm, pH 7.35). The extracellular solution contained: 140 mM NaCl, 3 mM KCl, 1.3 mM MgCl_2_, 2.4 mM CaCl_2_, 10 mM HEPES, and 10 mM glucose (298 mOsm, pH 7.4). Coverslips with transfected HEK293 cells were placed in a microchamber and continuously perfused with extracellular solution. ATP (Tocris Bioscience, Bristol, United Kingdom) was freshly prepared each day at 10 µM in extracellular solution and applied four times for 40 s, with 5 min washout intervals between applications. For each ATP application, the current trace during the stimulation period was extracted. Desensitization was calculated by expressing the current amplitude at the end of the 40 s of ATP application as a percentage of the maximum peak current reached during the same stimulus.

### 2.5 Immunofluorescence analysis

After 1 or 2 days of culture, TG neurons were washed with warm PBS for 5 min and fixed with 4% paraformaldehyde (PFA) and 4% sucrose in PBS at 37 °C for 20 min. Cells were then washed three times with PBS, permeabilized for 5 min with 0.2% Triton X-100 in PBS, and blocked with 5% bovine serum albumin (BSA) in PBS for 1 h at room temperature. Primary antibodies were diluted in 1% BSA and incubated overnight at 4 °C. The following antibodies and concentrations were used: anti-P2X2R rabbit (#APR-003, 1:300) and anti-P2X3R rabbit (#APR-016, 1:300) from Alomone Labs (Jerusalem, Israel); anti-CGRP mouse (#ab81887, 1:500) from Abcam (Cambridge, MA); anti-βIII tubulin mouse clone G7121 (1:1,000) from Promega (Madison, WI); anti-MAP1B goat N-19 (1:400) and anti-P2X2 goat V17 (1:100) from Santa Cruz Biotechnology (Dallas, TX). Isolectin B4 FITC conjugate (#L21895) from Sigma-Aldrich (St. Louis, MO) was used to label non-peptidergic neurons. After primary antibody incubation, coverslips were washed with PBS and incubated for 1 h at room temperature with the appropriate secondary antibodies: anti-rabbit Alexa Fluor 488 and anti-mouse Alexa Fluor 546 (Molecular Probes; Life Technologies, Grand Island, NY), in combination with DAPI (Thermo Fisher Scientific, Rockford, IL) to stain nuclei. Coverslips were then mounted using FluorSave mounting medium (Calbiochem, San Diego, CA) and imaged using a Zeiss LSM 710 Meta confocal microscope (Carl Zeiss Microscopy GmbH, Jena, Germany). Images were processed using LSM Image Browser software (Carl Zeiss Microscopy GmbH). Rat TG neuron cultures were classified by somatic size into three categories: small (<24 µm), medium (24–35 µm), and large (>35 µm) (Ono et al., 2012; Messlinger and Russo, 2019). In addition, mouse TG neurons cultured were classified as small (<15 µm), medium (15–25 µm), and large (>25 µm) (Simonetti et al., 2006; D’Arco et al., 2007). The somatic area (A) was measured by defining a region of interest (ROI) in ImageJ, and assuming circular geometry, the cell diameter (d) was calculated using the formula: $A=\pi r^{2}$ and d=2r .

### 2.6 Western blot analysis

Protein extracts from HEK293 cells or mouse TG were obtained using T-PER buffer (Pierce, Rockford, IL) supplemented with Complete Mini protease inhibitor cocktail tablets and PhosSTOP phosphatase inhibitor tablets (Roche Diagnostics, Indianapolis, IN). Protein concentrations were determined using the Bradford Protein Assay (Bio-Rad, Hercules, CA). Proteins were separated by SDS-PAGE and transferred to nitrocellulose membranes (Invitrogen, Carlsbad, CA). Membranes were blocked in 5% nonfat dry milk prepared in Tris-buffered saline with 0.05% Tween-20 (TBS-T) for 1 h at room temperature and then incubated overnight at 4 °C with primary antibodies diluted in 1% nonfat dry milk in TBS-T. After washing with TBS-T, membranes were incubated for 1 h at room temperature with the appropriate secondary antibodies, also diluted in 1% nonfat dry milk in TBS-T. Immunoreactive bands were detected using SuperSignal™ West Pico or Dura Chemiluminescent Substrate (Thermo Fisher Scientific, Rockford, IL). The following primary antibodies were used: anti-P2X2R rabbit (#APR-003, 1:1,000) and anti-P2X3R rabbit (#APR-016, 1:700) from Alomone Labs (Jerusalem, Israel); anti-GFP rabbit (#2555, 1:1,000) and anti-p35 (#2680, 1:500) from Cell Signaling Technology (Danvers, MA); anti-GAPDH (#MAB374, 1:4,000) from Merck Millipore (Darmstadt, Germany); anti-Na^+^/K^+^-ATPase α (H-3) (#sc-48345, 1:2,500) from Santa Cruz Biotechnology (Dallas, TX); and anti-α tubulin mouse (1:5,000) from (Sigma-Aldrich) and anti-βIII tubulin mouse clone G7121 (1:10,000) from Promega (Madison, WI). Band optical densities were quantified using ImageJ software, version 1.46 (NIH, Bethesda, MD).

### 2.7 Surface biotinylation assay

HEK293 cells transfected with P2X2aR, with or without co-transfection of the p35 vector, were washed twice with cold PBS (pH 8.0) and incubated with 0.4 mg/mL Sulfo-NHS-SS-Biotin (Pierce, Rockford, IL) in cold PBS (pH 8.0) for 45 min at 4 °C. The biotinylation reaction was quenched by adding Tris in PBS, pH 8.0 (TBS) to a final concentration of 25 mM. Cells were then washed twice with TBS and lysed to extract proteins. After protein quantification, 10% of the total protein extract was reserved for input controls, and the remaining 90% was incubated overnight at 4 °C with 40 μL of NeutrAvidin™ Agarose Resin (Pierce, Rockford, IL) in 0.5 mL PBS under gentle rotation. The mixture was centrifuged at 16,000 × g, and the pellet was washed three times with cold PBS to recover the surface-biotinylated proteins. Electrophoresis sample buffer was added to each sample to reach a final concentration of 2X. Samples were then incubated for 15 min at room temperature and heated at 70 °C for 10 min prior to analysis by Western blot.

### 2.8 Orofacial pain behavioral tests

#### 2.8.1 Orofacial thermal pain

Thermal sensitivity in the facial area was assessed in male and female mice as previously described (Neubert et al., 2005; Anderson et al., 2013). Cdk5 cKO mice in sensory neurons and their control littermates (4–8 months old), generously provided by Dr. Ashok Kulkarni (NIDCR, NIH) (Pareek et al., 2007), were deprived of food and water for 15 ± 1 h before testing. Mice were then placed in the Operant Orofacial Pain Assessment Device (OPAD; Stoelting Co., Wood Dale, IL). In this setup, thirsty mice must press their faces against dual Peltier elements, capable of reaching aversive temperatures, to access a reward bottle containing sugar-sweetened water. Mice were first trained at an innocuous temperature (33 °C) for 3 to 4 sessions in the OPAD device. During the following 2 weeks, each mouse was tested at either 33 °C or an aversive temperature of 45 °C, once daily for 10 min at one temperature per session. Data acquisition and analysis were performed using ANY-maze software, version 4.99 m (Stoelting Co., Wood Dale, IL). Thermal sensitivity was assessed by comparing total licking time at 33 °C versus 45 °C for each mouse. All behavioral tests were conducted at room temperature under natural light, between 9:30 and 12:00 h.

#### 2.8.2 Nociceptive behavior induced by intradermal injection of α,β-meATP

Pain-associated behaviors related to P2X3R and P2X2/3R activation were evaluated in male and female Cdk5 cKO mice and their littermate controls (aged 4–8 months). Behavioral tests were conducted in an acrylic observation box equipped with rear-facing mirrors and an external video camera. Each trial began with a 20-min acclimatization phase, during which mice were placed individually in the box. On the following day, mice received an intradermal injection of α,β-meATP, either into the hind paw (25–50 μL of 4–6 mM) or the whisker pad (25–50 μL of 5–8 mM), diluted in 0.9% saline. In some experiments, control animals received vehicle injections (0.9% saline). Immediately after injection, mice were returned to the observation box, and their behavior was recorded for 8 min using a video camera. Recordings were analyzed by an observer based on established nociceptive behaviors, including grooming and head flinching. All behavioral experiments were conducted at room temperature under artificial lighting, between 12:00 to 16:00 h.

### 2.9 Statistical analysis

All data are presented as mean ± SEM. Statistical analyses were performed using GraphPad Prism, version 7 (GraphPad Software, San Diego, CA). A p-value < 0.05 was considered statistically significant. Data were tested for normal distribution prior to analysis, and appropriate statistical tests were selected based on data distribution and experimental design. For comparisons between two groups, the Mann–Whitney test was used for Ca^2+^ imaging and surface biotinylation assays, and the Wilcoxon test was used for behavioral data. For comparisons involving more than two groups, two-way repeated measures ANOVA followed by Sidak’s multiple comparisons test was used for patch-clamp experiments. Mouse and rat τ_decay_ data were organized into histograms and analyzed using two-component Gaussian mixture models implemented in Python (Python Software Foundation; scikit-learn library) to classify the responses into distinct groups. The threshold separating fast and slow responses was defined as the τ_decay_ value at which the posterior probability of belonging to either Gaussian component was equal (P = 0.5 for each component).

## 3 Results

### 3.1 Immunolocalization of P2X2 and P2X3 in cultured murine TG neurons

**FIGURE 1:**
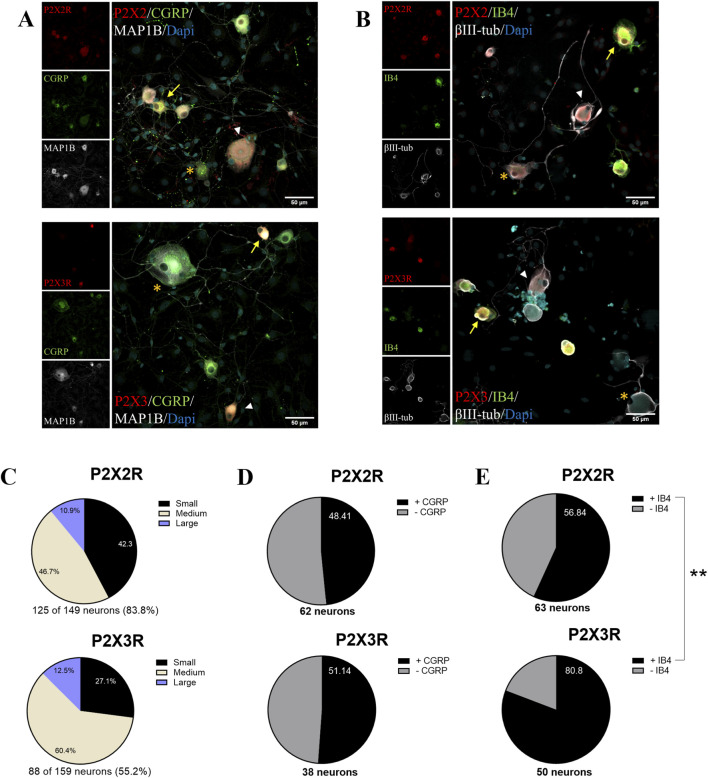
P2X2R and P2X3R channels are expressed in rat trigeminal neurons, including a substantial proportion of nociceptors. **(A)** Representative confocal images of primary culture of rat TG neurons immunostained for P2X2R or P2X3R (red), along with CGRP or **(B)** IB4 (green). Neurons were counterstained with βIII-tubulin or MAP1B (white) as neuronal markers and DAPI (cyan) to label nuclei. Yellow arrows indicate neurons co-expressing P2X2 or P2X3 with CGRP or IB4; white arrowheads indicate neurons positive for P2X2 or P2X3 but negative for CGRP or IB4; asterisks denote negative neurons for P2X2 or P2X3. **(C)** Pie charts show the distribution of small, medium, and large neurons among P2X2^+^ and P2X3^+^ populations in rat TG cultures. **(D, E)** Pie charts show the percentage of P2X2^+^ and P2X3^+^ neurons that are also positive for **(D)** CGRP and **(E)** IB4. Data is presented as percentage. **p < 0.01, Fisher’s exact test; n = 3 cultures. The number of neurons analyzed is indicated at the bottom of each graph. Scale bars: 50 mM.

Previously we demonstrated endogenous mRNA expression of P2X2a, P2X2b and P2X2e in mouse trigeminal ganglia (Coddou et al., 2017). Here, we performed primary cultures of rat TG neurons that were immunostained for P2X2 and P2X3 subunits (Figures 1A,B; Supplementary Figure S1A,S1B), as well as for Cdk5 and p35, which were detected in most neurons (Supplementary Figure S1C). Immunofluorescence analysis revealed that 83.8% and 55.2% of βIII-tubulin positive neurons express P2X2 and P2X3, respectively. These subunits were primarily found in small- and medium-sized trigeminal neurons (Figure 1C). Moreover, half of the P2X2 or P2X3 positive neurons (48.4% and 51.1%, respectively) also expressed CGRP, a neuropeptide marker for peptidergic nociceptors (Figures 1A,D) (Averill et al., 1995; Iyengar et al., 2017). In contrast, non-peptidergic nociceptors, stained against IB4 (Ambalavanar and Morris, 1992; Snider and McMahon, 1998), displayed a slightly different distribution, with 56.8% and 80.8% of P2X2- and P2X3-expressing neurons, respectively (Figures 1B,E). Similar results were obtained using primary cultures of mouse TG neurons (Supplementary Figures S2A–S2E). These findings suggest that most TG neurons expressing P2X2 or P2X3 in our primary cultures correspond to nociceptors.

### 3.2 Inhibition of Cdk5 induces a faster Ca^2+^ decay of α,β-meATP-evoked responses

**FIGURE 2:**
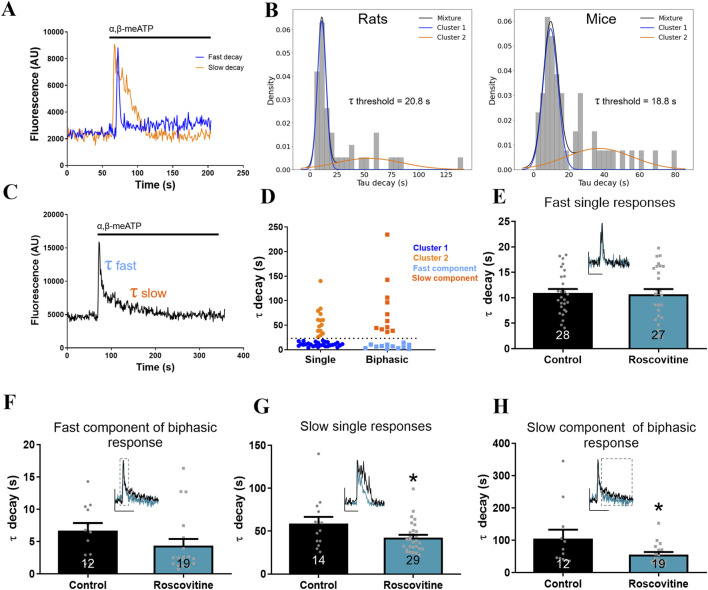
Roscovitine treatment accelerates Ca^2+^ decay in slow α,β-meATP-evoked responses in trigeminal neurons. **(A)** Representative trace showing fast (blue) and slow (orange) Ca^2+^ transients recorded in cultured TG neurons. α,β-meATP (100 µM) application is indicated by a black line. **(B)** Histograms of τ decay values from rat (left; n = 42) and mouse (right; n = 49) TG neurons. Gaussian mixture modeling identified two clusters with mean τ decay values of 10.8 s (blue) and 54.4 s (orange) in rat, and 9.6 s (blue) and 36.9 s (orange) in mouse. The τ decay threshold used to classify responses as fast or slow is indicated. **(C)** Representative trace of a biphasic Ca^2+^ transient showing both fast and slow τ decay components. α,β-meATP application is indicated by a black line. **(D)** Clustering analysis of τ decay values using Gaussian mixture models. A threshold (black dashed line) was defined based on the boundary between clusters 1 and 2. Fast and slow components of biphasic responses aligned with these clusters. **(E–H)** Quantification of τ decay kinetics in rat TG neurons treated with or without roscovitine (20 μM, 6 h), comparing: **(E)** fast single responses, **(F)** fast component of biphasic response, **(G)** slow single responses, and **(H)** slow component of biphasic responses. Insets in panels E–H show representative Ca^2+^ traces of TG neurons treated with vehicle (black line) and roscovitine (blue line). In panels F and H, dashed lines highlight the segments of the response corresponding to the fast and slow components. Data are presented as mean ± SEM. *p < 0.05, Mann–Whitney test; n = 3 cultures. The number of neurons analyzed is indicated below each bar.

To evaluate the activation of neurons expressing P2X3R and P2X2/3R, we performed Ca^2+^ imaging in murine TG primary cultures stimulated with the purinergic agonist α,β-meATP (100 µM). The resulting Ca^2+^ transients exhibited variable kinetics, which were fitted with single or double exponential decay functions to calculate τ_decay_, a constant that reflects the rate at which the signal returns to baseline. We observed two predominant τ_decay_ distributions in rat TG neurons (Figure 2A). To further analyze the distribution of τ_decay_ values, we applied Gaussian mixture modeling which revealed a good fit to two distinct populations. In rat TG neurons, the model identified groups with mean τ_decay_ values of 10.8 s (blue curve) and 54.4 s (orange curve), while in mouse TG neurons, the corresponding means were 9.6 s (blue curve) and 36.9 s (orange curve) (Figure 2B). Based on this distribution, we determined the τ_decay_ value corresponding to the intersection of the two Gaussian components and used this value as an objective threshold to classify responses as fast or slow. In rat Ca^2+^ transients, fast responses were defined as those with τ_decay_ < 20.8 s (43.1%), most likely driven by P2X3R activation, whereas slow responses were defined as τ_decay_ > 20.8 s (21.5%), predominantly associated with activation of P2X2/3R. Additionally, a subset of neurons exhibited biphasic responses (35.4%), characterized by an initial fast component followed by a slower decay phase (Figure 2C). Notably, both the fast and slow components of biphasic responses aligned with the corresponding single-response clusters (Figure 2D). These biphasic responses suggest co-expression of P2X3R and P2X2/3R within the same neurons. To evaluate whether Cdk5 modulates these responses, we treated cultured TG neurons with roscovitine (20 µM), a known Cdk5 inhibitor (Wang et al., 2005; Beaudry et al., 2015; Coddou et al., 2017; Sulak et al., 2018; Hwang and Namgung, 2021). Fast single responses and the fast component of biphasic responses showed no significant differences in τ_decay_ between roscovitine-treated and vehicle-treated neurons (Figures 2E,F), suggesting that Cdk5 inhibition does not affect the decay of these fast Ca^2+^ signals. In contrast, both slow single responses and the slow component of biphasic responses exhibited a significant reduction in τ_decay_ following roscovitine treatment (Figures 2G,H), indicating that Cdk5 inhibition accelerates the decay of these slower responses. This result supports the hypothesis that roscovitine selectively modulates responses associated with P2X2-containing receptors. Importantly, roscovitine did not alter the kinetics or amplitude of Ca^2+^ transients evoked by depolarization with 35 mM KCl (Supplementary Figure S3). In addition, nearly 95% of the neurons responding to α,β-meATP had small or medium soma sizes (Supplementary Figure S2E), supporting the notion that these responses primarily arise from nociceptors.

**FIGURE 3:**
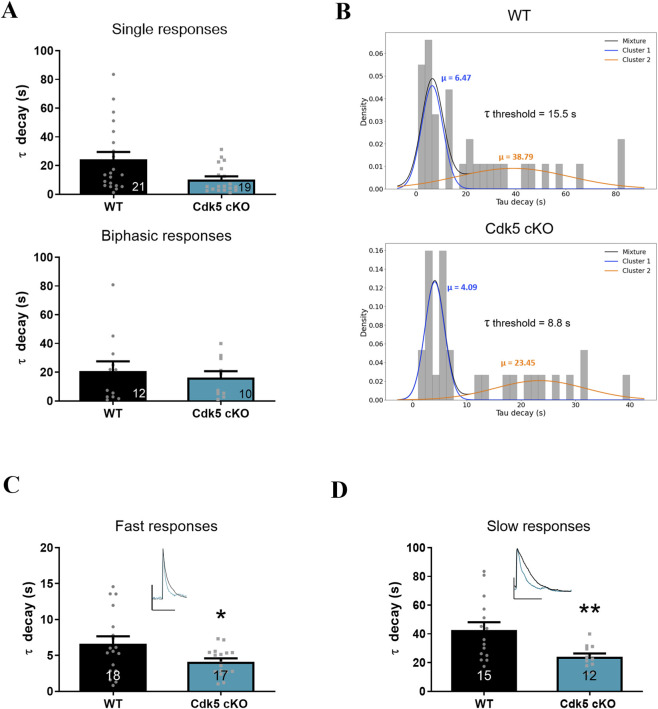
Conditional knockout of Cdk5 accelerates Ca^2+^ decay in α,β-meATP-evoked responses in mouse trigeminal neuron cultures. **(A)** Summary of τ decay values measured in TG neurons from Cdk5 cKO and WT mice. Single (top) and biphasic (bottom) responses are shown separately. **(B)** Histograms of τ decay values from WT (top; n = 33) and Cdk5 cKO (bottom; n = 29) TG neurons. Gaussian mixture modeling identified two clusters with mean τ decay values of 6.5 s (blue) and 38.8 s (orange) in WT, and 4.1 s (blue) and 23.5 s (orange) in Cdk5 cKO. The t decay threshold used to classify responses as fast or slow is indicated. **(C)** Quantification of τ decay kinetics classified as fast responses in mouse TG neurons comparing WT and Cdk5 cKO. **(D)** Quantification of τ decay kinetics classified as slow responses in mouse TG neurons comparing WT and Cdk5 cKO. Insets in panels C–D show representative Ca^2+^ traces of WT (black line) or Cdk5 cKO (blue line) TG neurons. Data are presented as mean ± SEM. *p < 0.05, **p < 0.01, unpaired t-test; n = 3 cultures. The number of neurons analyzed is indicated below each bar.

To complement these findings, we performed a Ca^2+^ imaging experiments from TG primary cultures from Cdk5 cKO and control littermates mice, in which Cdk5 was selectively deleted in sensory neurons (Pareek et al., 2007). Trigeminal neurons were stimulated by controlled perfusion of α,β-meATP (10 μM) to evaluate responses mediated by P2X3Rs and P2X2/3Rs. Both single and biphasic responses were identified in Cdk5 cKO and control TG cultures (Figure 3A). To increase statistical power for Gaussian mixture modeling, single and biphasic responses were combined due to the relatively small number of events. The resulting distributions again showed a good fit to two Gaussian components. Notably, the τ_decay_ distribution in Cdk5 cKO neurons was shifted toward lower values compared with WT cultures (Figure 3B). Consequently, the model-derived threshold separating fast and slow responses differed markedly between Cdk5 cKO (8.8 s) and WT (15.5) conditions. Importantly, slow responses exhibited a pronounced reduction in τ_decay_ in Cdk5 cKO cultures (Figure 3D), consistent with the effects observed following pharmacological inhibition of Cdk5 with roscovitine. In contrast, fast responses showed a smaller but statistically significant decrease in τ_decay_ (Figure 3C), suggesting that Cdk5 loss-of-function in this genetic model may influence the kinetics of both P2X3R- and P2X2/3R-mediated responses. Next, we studied the effect of pre-incubation with a selective antagonist of P2X3-containing receptors (A-317491) and we observed a marked reduction in the percentage of neurons responding to α,β-meATP-stimulation (from ∼8% in control conditions to <1% in treated cells), without affecting cell viability (Supplementary Figure S4). Additionally, we tested Gefapixant, another P2X3-selective antagonist, in rat primary cultures of TG neurons and we observed a markedly reduced α,β-meATP-evoked responses (from 21% to 2.4%) (Supplementary Figure S4). Altogether these results suggest that the responses evoked by α,β-meATP in TG neurons are mediated by P2X3 and P2X2/3 receptors.

### 3.3 Cdk5 modulates P2X2aR desensitization independently of receptor trafficking in HEK293 cells

**FIGURE 4:**
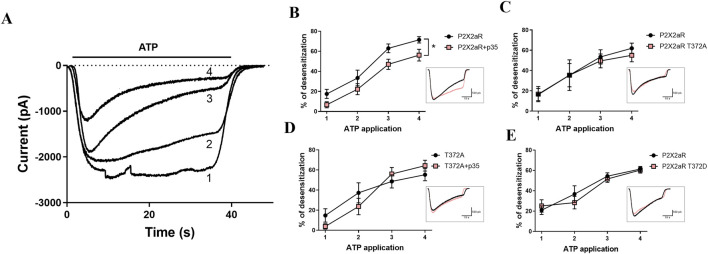
The T372A mutation in P2X2aR disrupts Cdk5-dependent modulation in HEK293 cells. **(A)** Representative whole-cell voltage-clamp recordings showing evoked currents after four consecutive applications of ATP (10 μM, 40 s each) in a HEK293 cell expressing P2X2aR. A 5 min washout interval separated each stimulus. The sequence of traces is labeled 1 to 4. **(B–E)** Quantification of desensitization percentages across four ATP applications in HEK293 cells expressing: **(B)** P2X2aR (n = 12) or P2X2aR + p35 (n = 15), **(C)** P2X2aR (n = 4) or P2X2aR T372A (n = 6), **(D)** P2X2aR T372A (n = 7) or P2X2aR T372A+ p35 (n = 4), and **(E)** P2X2aR (n = 11) or P2X2aR T372D (n = 11). Insets in panels B–E show representative current traces corresponding to the third ATP application under each condition. Holding potential: –60 mV. Data are presented as mean ± SEM. *p < 0.05, Two-way repeated measures ANOVA.

Since the P2X2a subunit is a target Cdk5 phosphorylation (Coddou et al., 2017), we further evaluate the implications of Cdk5-mediated phosphorylation at T372 on P2X2a functional properties. HEK293 cells that endogenously express Cdk5 were transfected with wild-type P2X2a, P2X2a mutants T372A (non-phosphorylatable) or T372D (phosphomimetic) in the presence or absence of p35, and we evaluated ATP-induced current desensitization using patch-clamp electrophysiology. HEK293 cells transfected were stimulated with four ATP pulses (10 µM), separated by 5 min washout intervals, to assess use-dependent desensitization (UDD) (Khadra et al., 2012; Coddou et al., 2015) (Figure 4A). Similarly to reported before (Coddou et al., 2017), co-transfection with p35 (activator of Cdk5) reduced UDD of the wild-type P2X2a which have a consensus sequence for Cdk5 phosphorylation (Figure 4B). In contrast, the T372A P2X2a mutant showed desensitization kinetics comparable to the wild-type P2X2a (Figure 4C), although did not respond to p35 co-expression (Figure 4D), confirming the relevance of this phosphorylation site for Cdk5-dependent modulation. We next tested whether mimicking phosphorylation of T372D P2X2a would reproduce the effect. Surprisingly, T372D P2X2a mutant showed no difference in UDD compared to wild-type P2X2a, suggesting that one negative charge alone is insufficient to mimic phosphorylation at this site (Figure 4E).

**FIGURE 5:**
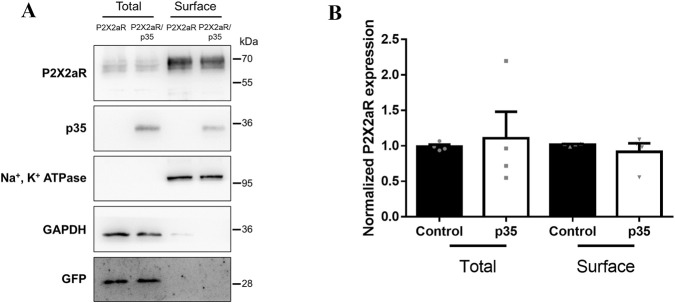
Activation of Cdk5 does not alter plasma membrane localization of P2X2aR in HEK293 cells. **(A)** Representative Western blot of total and surface protein fractions obtained by surface biotinylation assays in HEK293 cells transfected with P2X2aR-pIRES-GFP alone or co-transfected with p35. Immunodetection of P2X2aR, p35, Na^+^/K^+^-ATPase, GAPDH, and GFP is shown. Importantly, cytosolic markers such as GAPDH and GFP were largely absent from the surface fraction. In contrast, Na^+^/K^+^-ATPase marker is in surface fraction confirming successful separation. **(B)** Quantification of P2X2aR expression in total and surface protein fractions. Protein levels were compared between cells transfected with P2X2aR alone (control) or co-transfected with p35. P2X2aR was normalized to GAPDH in the total fraction and to Na^+^/K^+^-ATPase in the surface fraction. Data are presented as mean ± SEM; n = 4 independent experiments.

While altered channel biophysics may underlie the decreased desensitization observed with Cdk5 activity, we also considered the possibility that Cdk5 modulates other cellular processes, such as trafficking. Cdk5 has previously been implicated in regulating membrane trafficking, including the surface delivery of TRPV1 channels (Smith and Tsai, 2002; Xing et al., 2012; Liu et al., 2015; Liu et al., 2019). To test whether Cdk5 influences P2X2aR surface expression, we performed surface biotinylation assays in HEK293 cells transfected with P2X2aR-pIRES-GFP, with or without p35. After 24 h, biotinylated surface proteins were isolated, and P2X2aR surface levels, normalized to Na^+^/K^+^-ATPase, showed no significant difference with p35 overexpression (Figures 5A,B). Total P2X2aR protein levels, normalized to GAPDH, also remained unchanged. These results suggest that Cdk5-dependent regulation of P2X2aR desensitization in this model does not involve changes in receptor trafficking to or from the plasma membrane.

### 3.4 Cdk5 cKO mice exhibit reduced facial pain-like behavior evoked by α,β-meATP

**FIGURE 6:**
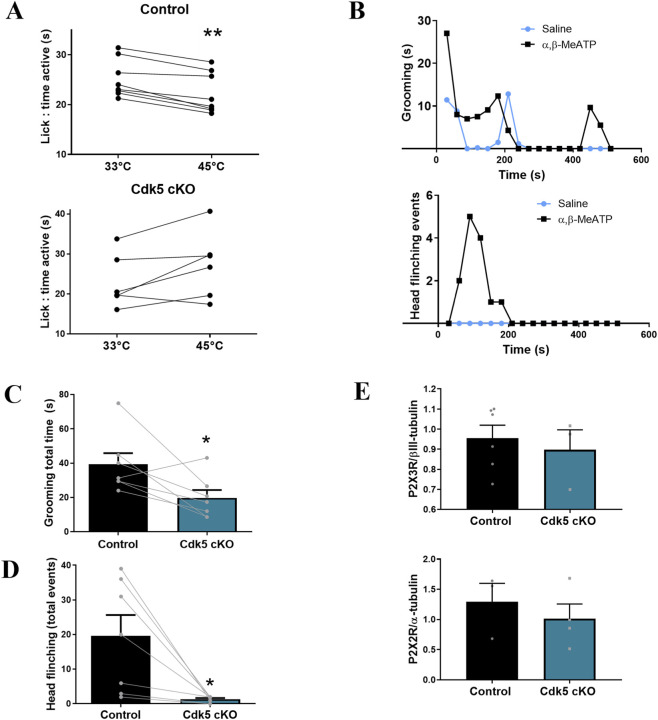
Cdk5 cKO mice exhibit reduced facial pain-like behavior following intradermal injection of α,β-meATP. **(A)** Time spent drinking at innocuous (33 °C) and noxious (45 °C) temperatures in the Orofacial Pain Assessment Device (OPAD) for control (top) and Cdk5 cKO (bottom) mice. Each pair of connected points represents individual mouse performance. Data represents the average of at least four test sessions. **p < 0.01, Wilcoxon test; n = 6–8 mice. **(B)** Representative time courses of grooming (top) and head flinching (bottom) behaviors measured every 30 s following intradermal injection of α,β-meATP (50 μL of 6 mM) or saline in two individual mice. **(C, D)** Quantification of **(C)** grooming total time (s) and **(D)** head flinching (total events) behaviors after intradermal injection of α,β-meATP (25 µL of 5–8 mM) in Cdk5 cKO and control littermates. Each pair of connected points represents a matched pair of control and Cdk5 cKO littermates. *p < 0.05, Wilcoxon test; n = 7 mice per genotype. **(E)** Western blots quantification of P2X3 and P2X2 protein levels in TG from control and Cdk5 cKO mice. βIII-tubulin and α-tubulin were used as loading control. Data are expressed as mean ± SEM; n = 3–6 independent experiments, as indicated.

To assess the role of Cdk5 in purinergic pain signaling, we evaluated α,β-meATP-evoked pain-like behavior in Cdk5 cKO mice, in which Cdk5 is selectively deleted in Nav1.8-expressing primary afferent neurons (Agarwal et al., 2004). These mice have previously been reported to display hypoalgesia to noxious heat stimuli (Pareek et al., 2007; Jendryke et al., 2016). We first validated the Cdk5 cKO model using the OPAD system, which measures orofacial thermosensitivity based on licking behavior at innocuous (33 °C) and aversive (45 °C) temperatures. Littermates control mice showed a significant reduction in licking sugar-sweetened water at 45 °C as compared with 33 °C, consistent with thermal avoidance behavior. In contrast, Cdk5 cKO mice showed no difference in licking between the two temperatures (Figure 6A), indicating a blunted response to thermal nociception and confirming that Cdk5 deletion in sensory neurons decreased pain behavior. To evaluate nocifensive responses to α,β-meATP, we first verified that intraplantar injection of the agonist (4–6 mM) into the hind paw of control mice evoked robust pain-like behaviors, including paw licking and lifting (data not shown), consistent with previous reports (Bland-Ward and Humphrey, 1997; Hamilton et al., 1999). However, α,β-meATP-induced facial pain responses have been less studied. To address this, we injected awake control mice with α,β-meATP or vehicle (0.9% saline) into the whisker pad and videorecorded their behavior for later analysis. We observed spontaneous nociceptive behaviors such as facial grooming and head flinching, a sudden head-and-body movement into a stooped posture followed by a return to the normal standing position. We found that α,β-meATP injection increment time of grooming (s) and events of head flinching as compared with vehicle injection (Figure 6B). Then, we injected α,β-meATP into the whisker pad of 6 pairs of Cdk5 cKO and control littermates and compared these nociceptive responses within each pair. We found that Cdk5 cKO mice exhibited significantly fewer grooming and head flinching episodes as compared with control mice (Figures 6C,D), indicating reduced facial pain-like behavior in response to purinergic stimulation. Importantly, P2X3 and P2X2 protein levels (Figure 6E) in the trigeminal ganglia were similar between Cdk5 cKO and control mice, suggesting that the behavioral differences were not due to altered receptor expression.

## 4 Discussion

In this study, we demonstrate that Cdk5 modulates purinergic signaling in trigeminal nociceptors using complementary pharmacological and genetic loss-of-function approaches. We found that pharmacological inhibition or conditional deletion of Cdk5 accelerated the decay of α,β-meATP–evoked Ca^2+^ transients, with a predominant effect on slow responses. Moreover, facial pain-like behavior evoked by α,β-meATP was significantly reduced in Cdk5 cKO mice, suggesting that Cdk5 modulation of P2X3R and P2X2/3R may have physiological relevance in orofacial pain signaling. Importantly, our findings extend previous observations of Cdk5-dependent modulation of ion channels by identifying purinergic P2X2-containing receptors as a novel functional target in trigeminal nociceptors. This expands the repertoire of Cdk5-regulated pathways in pain signaling beyond TRP channels and highlights purinergic signaling as a key mechanistic node.

Phosphorylation is a well-established regulatory mechanism for P2XRs in ATP-mediated signaling (Coddou et al., 2011). α,β-meATP is a selective agonist commonly used to study sensory neuron responses, as it activates P2XRs containing P2X1 or P2X3 subunits (Dunn et al., 2001; North, 2002). In our study, this agonist elicited Ca^2+^ transients with distinct kinetic profiles, reflecting fast and slow desensitization kinetics, as previously reported in heterologous systems (Koshimizu et al., 2000; He et al., 2003). Since P2X1 has not been shown to play a functional role in sensory neurons (Rae et al., 1998; Cockayne et al., 2000; Zhong et al., 2001; Shinoda et al., 2005), and α,β-meATP does not activate P2X2 or P2X4 homotrimers at the concentrations used in our study (Gever et al., 2006), the fast and slow responses we observed most probably correspond to activation of P2X3R and P2X2/3R (Cook et al., 1997; Cockayne et al., 2005; Simonetti et al., 2006; Ye et al., 2014). Biphasic Ca^2+^ transients observed in our study are consistent with electrophysiological evidence from both DRG and TG neurons, where α,β-meATP-evoked currents show biphasic decay due to the co-expression of multiple configurations of P2X3 and P2X2 subunits within the same cell (Burgard et al., 1999; Grubb and Evans, 1999; Simonetti et al., 2006; D’Arco et al., 2007). Importantly, Cdk5 loss-of-function in our *in vitro* models primarily affected the slow components of Ca^2+^ responses, both in biphasic and single-transient profiles. Cumulative evidence suggests that these effects are most consistent with modulation of P2X2a-containing receptors, which exhibit slower desensitization kinetics and are known targets of Cdk5 phosphorylation. Our previous work demonstrated that Cdk5 phosphorylates the P2X2a subunit at T372, slowing the decay of P2X2aR currents but not P2X2bR or P2X2eR, which lack the Cdk5 consensus phosphorylation site (Coddou et al., 2017). Notably, P2X3R does not contain a canonical Cdk5 phosphorylation consensus site. Nevertheless, substantial evidence indicates that P2X3R activity is regulated by multiple interacting proteins that influence receptor phosphorylation state, plasma membrane localization, recovery, and protein–protein interactions (Fabbretti, 2013). In addition, these regulatory pathways can themselves be modulated by upstream signaling proteins, including Cdk5 (Nair et al., 2010a; Nair et al., 2010b). Based on the results shown on Figure 3C, it is possible that Cdk5 loss-of-function alters sensory neuron physiology and indirectly accelerates P2X3R kinetics through such regulatory mechanisms. Interestingly, pharmacological inhibition of Cdk5 for 6 h did not modify fast responses, suggesting that this modulation may occur through longer-term processes rather than through acute kinase inhibition.

Alternatively, Cdk5 inhibition may affect other mechanisms that influence intracellular Ca^2+^ levels. P2XR activation can lead to the opening of voltage-gated calcium channels (VGCCs) (Gu and MacDermott, 1997; Koshimizu et al., 2000; Song et al., 2007), and Cdk5 has been shown to modulate several VGCCs (Tomizawa et al., 2002; Yan et al., 2002; Su et al., 2012; Furusawa et al., 2014). Additionally, Ca^2+^ decay kinetics depend on extrusion and buffering mechanisms (Gover et al., 2007). While previous electrophysiological studies have linked slow α,β-meATP-evoked responses to P2X2R-containing receptors in sensory neurons in a direct manner (North, 2002; Simonetti et al., 2006; D'Arco et al., 2007), it is important to note that the Ca^2+^ dynamics not only reflect the activity of α,β-meATP-gated receptors responsible for Ca^2+^ influx, but are also shaped by downstream intracellular buffering and extrusion processes. However, if Cdk5 inhibition affected these global mechanisms, we would expect to see broader changes in Ca^2+^ signaling during KCl-induced depolarization, which were not observed in our experiments.

Our electrophysiological data in HEK293 cells confirmed previous findings that Cdk5 reduces P2X2aR desensitization. The T372A mutant, which lacks the Cdk5 phosphorylation site, failed to respond to p35 co-expression, indicating that phosphorylation at this residue is essential for Cdk5-mediated modulation. The phosphomimetic T372D mutant did not reproduce the effects of phosphorylation, suggesting that a single negative charge introduced by the aspartate substitution is insufficient to fully mimic the electrostatic and structural properties of a phosphate group. Future work should focus on generating alternative phosphomimetic mutants to more effectively replicate the effect of phosphorylation and better understand its role in P2X2a desensitization.

We also investigated whether Cdk5 could regulate P2X2aR surface expression, given its known role in trafficking and cytoskeletal dynamics (Smith and Tsai, 2002; Kawauchi, 2014). However, surface biotinylation assays showed no change in P2X2aR surface levels upon p35 co-expression, suggesting that Cdk5’s effect on receptor function in this model is due to changes in receptor biophysics rather than trafficking. This is consistent with the idea that P2X2R, unlike P2X1R, P2X3R, or P2X4R, is relatively stable at the plasma membrane (Robinson and Murrell-Lagnado, 2013).

Cumulative evidence place P2X3R and P2X2/3R as the principal afferent transducers of ATP signaling during acute and inflammatory pain (Hamilton et al., 1999; Barclay et al., 2002; Wu et al., 2004; Cockayne et al., 2005; Oliveira et al., 2005; Adachi et al., 2010; Teixeira et al., 2010; Watanabe et al., 2010), also leading to the development of pain hypersensitivity (Hamilton et al., 1999; Tsuda et al., 2000; Shinoda et al., 2005; Maruyama et al., 2018) or even chronic pain (Bernier et al., 2018). While peripheral P2X3R or P2X2/3R activation in the hind paws is well studied, orofacial pain has received less attention (Shinoda et al., 2005; D’Arco et al., 2007; Shinoda et al., 2008; Adachi et al., 2010). In our model, the intradermal injection of α,β-meATP into the whisker pad elicited two well-stablished indicators of spontaneous pain in rodents: facial grooming and head flinching (Krzyzanowska and Avendano, 2012; Deuis et al., 2017). Head flinching has been previously observed in models of temporomandibular joint inflammation (Roveroni et al., 2001; Oliveira et al., 2005; Rodrigues et al., 2006; Bonjardim et al., 2009; Teixeira et al., 2010; Oliveira-Fusaro et al., 2012; Melo et al., 2017) and in response to noxious stimulation of the masseter muscle (Shinoda et al., 2008). As expected, this behavior was not observed after saline injection (Roveroni et al., 2001; Bonjardim et al., 2009; Melo et al., 2017), supporting its specificity for painful stimuli. On the other hand, facial grooming was observed at baseline and after saline injection, likely due to its broader role in non-nociceptive functions such as thermoregulation and stress responses (Leonard et al., 2005; Smolinsky et al., 2009; Almeida et al., 2015). Nevertheless, both behaviors have been associated with orofacial activation of P2X3R and P2X2/3R (Oliveira et al., 2005; D’Arco et al., 2007; Shinoda et al., 2008; Teixeira et al., 2010), suggesting that stimulation of fibers expressing these receptors is sufficient to trigger pain-like responses. Interestingly, facial grooming and head flinching were significantly lower following α,β-meATP injection in Cdk5 cKO mice as compared with control mice, indicating a critical role for Cdk5 in modulating purinergic pain responses in the orofacial region. These results in mouse models suggest that Cdk5-dependent phosphorylation of P2X2-containing purinergic channels could be of physiological and pathological relevance. Importantly, P2X3 and P2X2 protein levels in the trigeminal ganglia were not altered between genotypes, suggesting that the behavioral phenotype was not due to receptors expression downregulation. The combination of our behavioral assay and the genetic model enabled more refined observations. On one hand, we focused on spontaneous responses evoked by an agonist that selectively targets a limited subset of P2XRs, avoiding the need for additional stimuli that might engage other signaling pathways. On the other hand, our genetic model restricts Cdk5 deletion to a specific population of sensory neurons, primarily nociceptors, allowing for a more targeted analysis of its role in purinergic pain signaling. These findings support the idea that Cdk5 regulates transmission through P2X3R- and/or P2X2/3R-expressing nociceptors, a mechanism that may be physiologically relevant in the context of acute or inflammatory pain. Indeed, enhancing receptor desensitization has been proposed as a potential therapeutic approach for analgesia (Giniatullin and Nistri, 2013).

In summary, our results demonstrate that loss of Cdk5 function alters α,β-meATP-evoked Ca^2+^ dynamics and reduces pain-like behavior, indicating a basal regulatory role for Cdk5 in P2X-mediated nociception. Data from the OPAD system further suggest that Cdk5 contributes to acute thermosensitivity to noxious heat (Pareek et al., 2007; Jendryke et al., 2016). Given that heat sensing relies on redundant signaling from TRPV1, TRPA1, and TRPM3 (Vandewauw et al., 2018), and that two of these channels (TRPV1 and TRPA1) are known Cdk5 substrates (Pareek et al., 2007; Hall et al., 2018; Sulak et al., 2018), it is reasonable to speculate that Cdk5 plays a broader role in setting nociceptive thresholds. Over the past decade, Cdk5 has emerged as a key modulator of peripheral nociceptive signaling. Our findings suggest that Cdk5 basally modulates orofacial pain mediated by P2X3R and P2X2/3R. According to our findings, this mechanism may be due to the phosphorylation of the P2X2a subunit, suggesting that P2X2/3R represents a physiologically relevant substrate of Cdk5, with important implications for orofacial pain signaling. Although our results strongly support a model in which Cdk5 phosphorylates P2X2a to modulate ATP-evoked desensitization, further work is needed to directly demonstrate this modification in native sensory neurons. Phosphoproteomic profiling of the trigeminal ganglia could provide direct biochemical validation. In addition, generating knock-in mice carrying the non-phosphorylatable T372A mutation would represent a critical step toward confirming the physiological significance of this residue *in vivo*. These tools would also help to assess whether Cdk5-mediated phosphorylation integrates with scaffolding or adaptor proteins to stabilize receptor complexes at nociceptive terminals.

## Data Availability

The original contributions presented in the study are included in the article/Supplementary Material, further inquiries can be directed to the corresponding authors.
