# Supplementary material for: Cdk5 modulates orofacial pain through P2X2/3 purinergic receptor-mediated signaling in trigeminal neurons
Source: Front Pharmacol. 2026 May 4;17:1767160. doi: 10.3389/fphar.2026.1767160 (PMC13180896; doi:10.3389/fphar.2026.1767160)
Supplement: Supplementary file 1 [file Supplementaryfile1.docx]

Cdk5 modulates orofacial pain through P2X2/3 purinergic receptor-mediated signaling in trigeminal neurons

Supplementary Material

**Supplementary Figures**


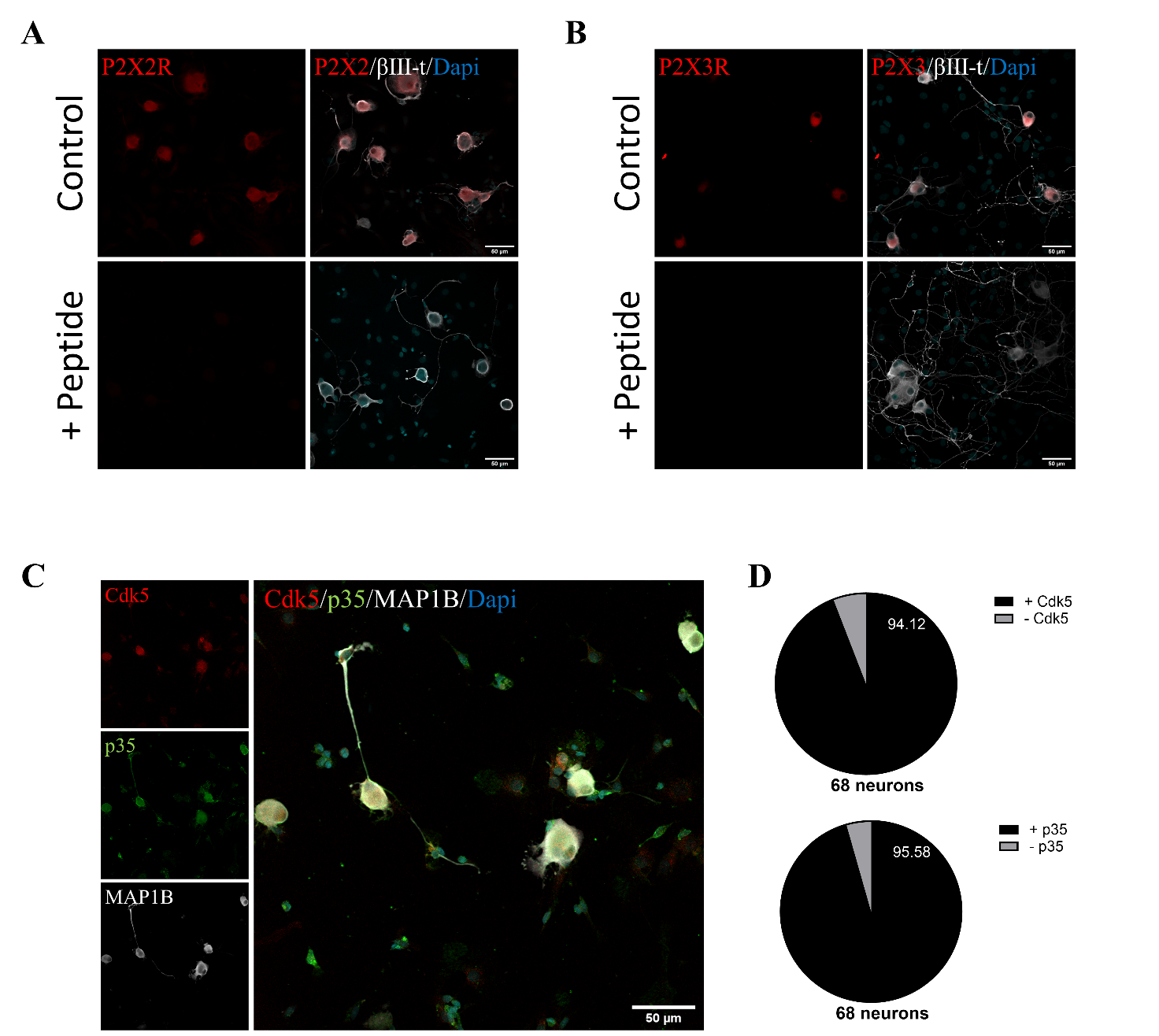


**Supplementary Figure S1. Validation of P2X2 and P2X3 antibodies specificity and expression of Cdk5 and p35 in rat TG cultures**. Representative confocal images of primary culture of rat TG neurons immunostained for **(A)** P2X2 and **(B)** P2X3 (red) in the absence (top panels) or presence (bottom panels) of the corresponding control peptide antigen for P2X2 and P2X3. TG neurons were labeled with βIII-tubulin (white). **(C)** Representative confocal image showing immunostaining for Cdk5 (red) and p35 (green). Both proteins are primarily localized to neurons, identified by MAP1B immunostaining (white). **(D)** Pie charts showing the percentage of MAP1B^+^ neurons that are also positive for Cdk5 (top) and p35 (bottom). Data are expressed as percentage. n = 3 cultures. The number of neurons analyzed is indicated below each pie chart.

**
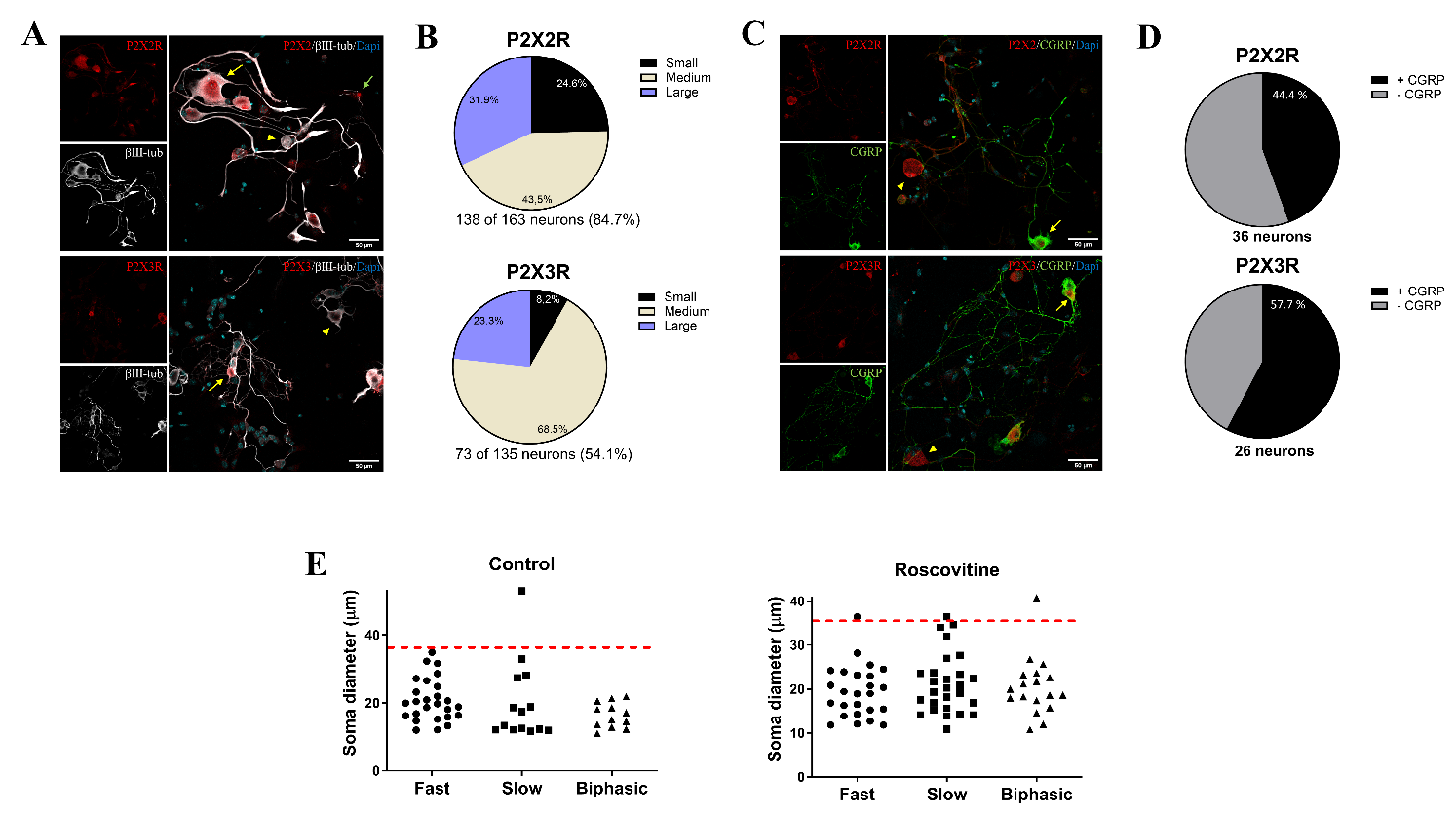
**

**Supplementary Figure S2. Expression of P2X2 and P2X3 in high proportion of nociceptors in primary culture of mouse TG neurons.** **(A)** Representative confocal images of primary culture of mouse TG neurons immunostained for P2X2 and P2X3 (red). Neurons were labeled with βIII-tubulin (white), and cell nuclei were stained with DAPI (cyan). Yellow arrows indicate P2X2+ or P2X3+ neurons. Yellow arrowheads indicate P2X2- or P2X3- neurons. Green arrow indicates a βIII-tubulin- expressing P2X2 (non-neuronal cell). **(B)** Pie charts showing the distribution of small, medium, and large neurons among P2X2+ and P2X3+ populations in mouse TG cultures (n = 3 cultures). **(C)** Representative confocal image showing P2X2 or P2X3 (red) co- immunostained with CGRP (green). Nuclei are stained with DAPI (cyan). Yellow arrows indicate neurons co-expressing P2X2 or P2X3 with CGRP; yellow arrowheads indicate neurons lacking CGRP expression. **(D)** Percentage of P2X2+ and P2X3+ neurons that are also CGRP+. n = 2 cultures. The number of neurons analyzed is indicated below each pie chart. **(E)** Soma diameter of neurons for TG neurons previously classified that performed fast-, slow-, or biphasic-responses to α,β-meATP. The red dotted line marks the boundary between small/medium and large neurons based on soma size.

**
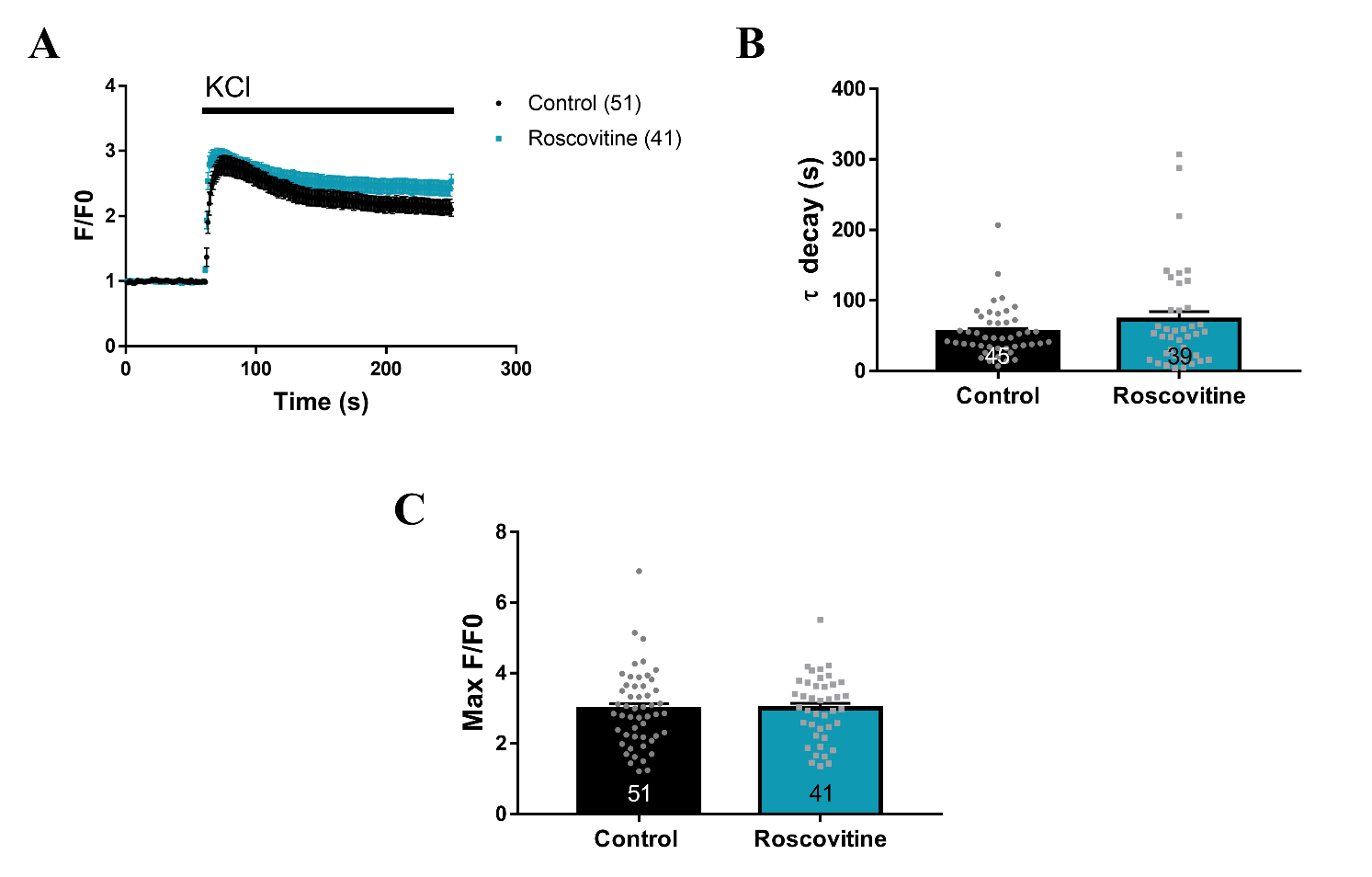
**

**Supplementary Figure S3. Roscovitine does not alter Ca2+ responses evoked by KCl-induced depolarization in rat TG cultures.** **(A)** Summary of KCl-evoked Ca2+ responses in TG primary cultures under control conditions (black line) or after roscovitine treatment (light blue line). The application of KCl (35 mM) is indicated by a black bar. **(B, C)** Quantification of **(B)** τ decay and **(C)** peak amplitude in control and roscovitine-treated neurons. For τ decay analysis, responses were fitted to a single exponential function; 6 out of 51 control traces and 2 out of 41 roscovitine traces were excluded due to poor fit. No significant differences were observed in either parameter. Data are presented as mean ± SEM; n = 2 cultures. The number of neurons analyzed is indicated below each bar.

**
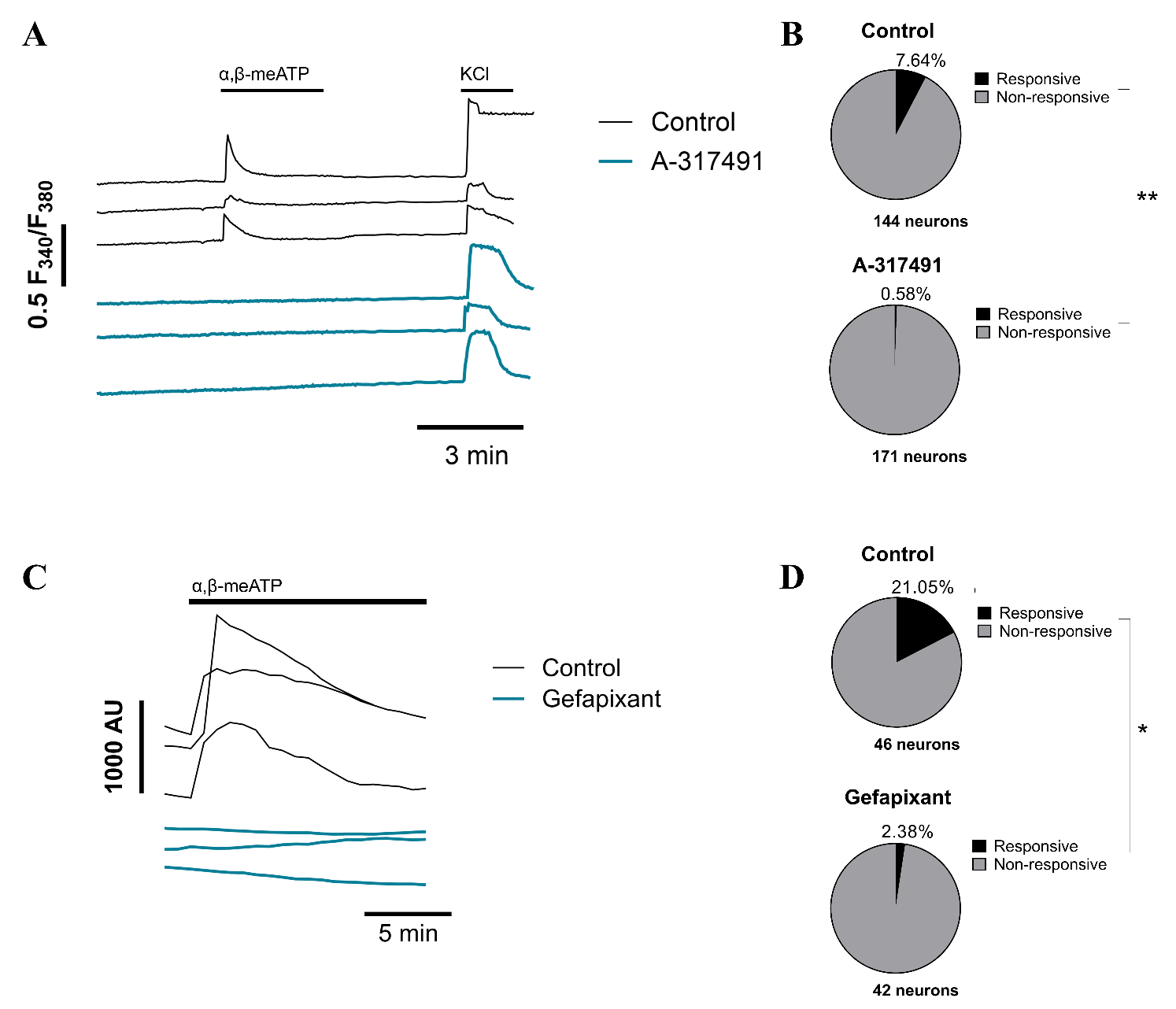
**

**Supplementary Figure S4. The P2X3R and P2X2/3R antagonists A-317491 and gefapixant inhibit α,β- meATP-evoked responses in mouse and rat TG cultures.** **(A)** Representative Ca^2+^ traces from mouse TG neurons showing responses to α,β-meATP under control conditions and after incubation with A-317491. Three representative examples are shown for each condition. The timing of α,β- meATP (10 μM) and KCl (30 mM) application is indicated by the black bar. **(B)** Pie charts summarize the proportion of α,β-meATP-responsive and non- responsive neurons in control and A-317491-treated cultures. Treatment with A-317491 significantly reduced the proportion of responsive neurons compared to control (**p < 0.01, Fisher’s exact test), without affecting cell viability, as assessed by the proportion of KCl-responsive neurons (control: 89% [144/161] vs A-317491: 88% [171/194]). **(C)** Representative Ca^2+^ traces from rat TG neurons showing responses to α,β-meATP under control conditions and after incubation with gefapixant. Three representative examples are shown for each condition. The timing of α,β-meATP (100 μM) application is indicated by the black bar. **(D)** Pie charts summarize the proportion of α,β-meATP-responsive and non-responsive neurons in control and gefapixant-treated cultures. Treatment with gefapixant significantly reduced the proportion of responsive neurons compared to control (*p < 0.05, Fisher’s exact test).
